# Cerebral Perfusion Insufficiency and Relationships with Cognitive Deficits in Alzheimer’s Disease: A Multiparametric Neuroimaging Study

**DOI:** 10.1038/s41598-018-19387-x

**Published:** 2018-01-24

**Authors:** Chi-Wei Huang, Shih-Wei Hsu, Ya-Ting Chang, Shu-Hua Huang, Yung-Cheng Huang, Chen-Chang Lee, Wen-Neng Chang, Chun-Chung Lui, Na-Ching Chen, Chiung-Chih Chang

**Affiliations:** 1 Department of Neurology, Cognition and Aging Center, Kaohsiung Chang Gung Memorial Hospital, Chang Gung University College of Medicine, Kaohsiung, Taiwan; 2 Department of Radiology, Kaohsiung Chang Gung Memorial Hospital, Chang Gung University College of Medicine, Kaohsiung, Taiwan; 3 Department of Nuclear Medicine, Kaohsiung Chang Gung Memorial Hospital, Chang Gung University College of Medicine, Kaohsiung, Taiwan; 4 Department of Radiology, Division of medical imaging, E-Da Cancer Hospital and I-Shou University, Kaohsiung, Taiwan

## Abstract

Micro- or macro-circulatory insufficiency has a negative impact in patients with Alzheimer’s disease (AD). This study used arterial spin-labeled magnetic resonance imaging (ASL-MRI) and ethylcysteinate dimer single-photon emission computed tomography (ECD-SPECT) in 50 patients with AD and 30 age-matched controls to investigate how hypoperfusion patterns were associated with gray matter atrophy and clinical data. All participants completed 3DT1-MRI, ECD-SPECT and ASL-MRI examinations. Medial temporal cortex (MTC) volumes were correlated with regional signals showing significantly lower relative cerebral blood flow (rCBF) in ASL-MRI or perfusion index (PI) in ECD-SPECT. Neurobehavioral scores served as the outcome measures. Regions with lower PI showed spatial similarities with atrophy in the medial, anterior and superior temporal lobes, posterior cingulate cortex and angular gyrus, while regions showing lower rCBF were localized to the distal branches of posterior cerebral artery territories (posterior parietal and inferior temporal lobe) and watershed areas (angular gyrus, precuneus, posterior cingulate gyrus and middle frontal cortex). rCBF values in watershed areas correlated with MTC volumes and language composite scores. Precuneus and angular gyrus hypoperfusion were associated with the corresponding cortical atrophy. Macro- or micro-vasculature perfusion integrities and cortical atrophy determined the overall perfusion imaging topography and contributed differently to the clinical outcomes.

## Introduction

In late-onset Alzheimer’s disease (AD), atrophy of the medial temporal cortex (MTC) and posterior parietal cortex are early structural changes^1^, while amyloid toxicity^2^ is generally thought to underlie the degenerative mechanism. The subsequent appearance of synapse loss, amyloid plaques and neurofibrillary tangle formation^2^, may then lead to the onset of cognitive impairment. The presence of vascular risk factors^3^ and advanced aging process may then act in combination with the pathological processes to trigger cerebral macro- or micro-circulatory disturbances^4^.

Arterial spin labeling-magnetic resonance imaging (ASL-MRI)^5^ and ethyl cysteinate dimer single-photon emission computed tomography (ECD-SPECT)^6^ both measure cerebral perfusion. In ASL-MRI, magnetically labeled arterial blood serves as a diffusible endogenous tracer to quantify perfusion, thereby avoiding the need for injections and exposure to ionizing radiation^5^. ASL-MRI findings have been shown to be closely correlated with those of ^15^O-water positron emission tomography^7^, however study also showed that ASL-MRI systematically underestimated CBF especially in voxels supplied by two arteries^8^. Furthermore, morphological information can be obtained with good spatial resolution by means of three-dimensional (3D) T1 images. The most consistent finding in the literature of ASL-MRI applied to AD is a decreased CBF in the precuneus, angular gyrus and posterior cingulate cortex (PCC)^9^. A recent report suggested that the default mode network may be comprised of multiple, spatially dissociated but interactive components, of which two subsystems are particularly relevant: the “medial temporal lobe subsystem”, and the “dorsal medial prefrontal cortex subsystem” (or the midline core subsystem)^10^. Both PCC and precuneus areas have been reported to represent important cortical hubs of the midline core subsystem^11^.

The limitations of ECD-SPECT in quantifying perfusion include low spatial resolution and the lack of absolute values^12^, however signal retention has been shown to be highly parallel to regional CBF^13^. In the diagnostic criteria of AD^14^ ECD-SPECT is considered to be a reliable clinical biomarker reflecting neuronal injury. However, the role of ASL-MRI has yet to be clarified, and comparisons with ECD-SPECT may help to elucidate its diagnostic repertoire. Reported levels of agreement among cortical areas between MRI and SPECT perfusion are highly variable^15,16^.

The top-down modulation theory between posterior brain network hypoperfusion and MTC cortical atrophy is still under debate. One theory supports upstream hippocampal atrophy and downstream posterior parietal cortical hypoperfusion^17^, whereas another postulates that vascular damage and reduced perfusion in the parietal association cortex, PCC and precuneus lead to the initiation and aggravation of AD pathology in the MTC^18^. Although the metabolic changes of default mode network and medial temporal atrophy are well known, multi-parametric modalities using ECD-SPECT and ASL-MRI with structural comparisons to delineate the top-down modulation theory in patients with AD are still lacking.

Based on the hypothetical models of dynamic biomarkers in AD^17^, the study explored the topography similarities and differences among 3 imaging modalities, ASL-MRI, 3DT1-MRI and SPECT. By regression models, we proposed possible mechanisms for the mismatch of cerebral hypoperfusion and gray matter (GM) atrophy. The impact of reduced CBF and cognitive scores were used to validate the clinical significance of vascular risk factors.

## Materials and Methods

This study was conducted in accordance with the Declaration of Helsinki and was approved by the Institutional Review Board of Chang Gung Memorial Hospital. The study participants and their caregivers agreed on the participation of the study with written informed consent. The study participants were treated at the Cognition and Aging Center, Department of General Neurology, Kaohsiung Chang Gung Memorial Hospital. A total of 50 patients with AD and 30 healthy age-matched controls were included after the consensus of a panel composed of neurologists, neuropsychologists, neuroradiologists and experts in nuclear medicine^19^. AD was diagnosed according to the International Working Group criteria^20^ with a clinical diagnosis of typical AD. All of the patients had a clinical dementia rating (CDR) score of 0.5 or 1. The age-matched controls were selected from a normal cohort database, and all had normal cognition and basic biochemical profiles^21^. All of the patients with AD in this study received acetylcholine esterase inhibitors as standard treatment. The exclusion criteria were a past history of clinical stroke, a modified Hachinski ischemic score >4^22^ and moderate to severe stenosis (>50% stenosis) of major extra- or intra-cranial arteries by Doppler^23^. Patients with vascular stenosis or stroke were excluded because their asymmetric cerebral perfusion profile may have outweighed the degenerative process and confounded the results.

### Clinical and cerebral vascular risk factors

After enrolment, the demographic data of each patient were recorded. Cerebral vascular risk factors included age, high sensitivity C-reactive protein (hsCRP), total cholesterol, triglycerides, high-density lipoprotein (HDL), low-density lipoprotein, creatinine, and the presence of diabetes mellitus^24^ and hypertension^25^. The genotype of apolipoprotein E (APOE) was determined by polymerase chain reaction-restriction fragment length polymorphism assay and restriction enzyme HhaI^19^. APOE4 carriers were defined as those with one or two APOE4 alleles.

### Neurobehavioral assessments

A trained neuro-psychologist administered the neurobehavioral tests. The Mini-Mental State Examination (MMSE)^26^ and Cognitive Abilities Screening Instrument (CASI)^27^ total scores were used as a global assessment of cognitive function. In the CASI, the subdomain scores of verbal fluency and language were summarized as the language composite score to reflect the general language skill.

### Image acquisition

#### 3DT1 and ASL-MRI

MRI was performed on a 1.5T scanner (Discovery MR450, GE Healthcare, Milwaukee, WI) with an 8-channel head coil. High-resolution T1-weighted images of the whole brain anatomy were acquired using 3D spoiled gradient-recalled acquisitions in steady state sequences with the following parameters: repetition time/echo time/flip angle = 8.616 ms/4.2 ms/12°. One hundred and sixty sagittal slices covering the whole brain were acquired in 9 minutes and 14 seconds, with an isotropic spatial resolution of 1 mm^3^.

Resting CBF measurements were acquired using a pseudocontinuous ASL technique^28^ with a background-suppressed 3D fast spin echo sequence. The imaging parameters were as follows: TR/TE/label time/post-label delay = 4652/10.5/1500 /1525/ms, voxel size = 3.64 × 3.64 × 4 mm^3^, slice thickness = 4 mm, number of excitations = 3, number of slices = 38 and a scan time of 4 minutes to cover the whole brain. The study protocol followed that by Sigurdsson *et al*.^29^ that showed reliability for geriatric population at 1.5 Tesla MRI. The exclusion of patients with vascular stenosis was to reduce the possibility of prolonged arterial transit time. We didn’t use the vascular crushing gradients because it would reduce the signal-to-noise ratio. Besides, in normal situation, the labeled bolus will be delivered to target tissues and little labeled blood will be in large arteries at the time of imaging. In contrast, if extremely prolonged arterial transit time, bright vascular signals could be removed using vascular crushing gradients, but the quantitative CBF values in areas distal to the large vessel are error^30^.

**Figure 1:**
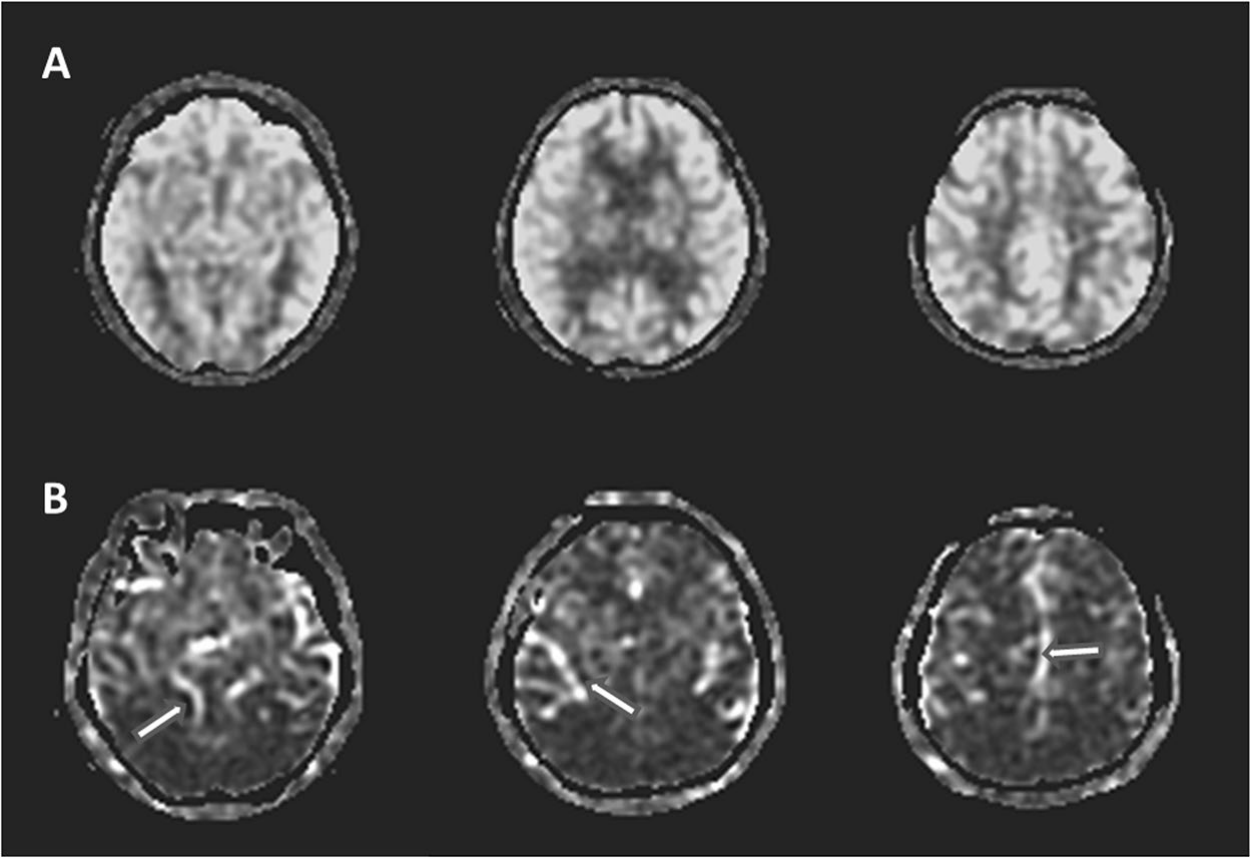
Raw arterial spin labeling (ASL) images of the represented subjects. (**A**) ASL perfusion images of a 70 year-old subject that is eligible for image quality checkup and enrollment. (**B**) ASL perfusion images from a 72 year-old subject that showed proximal portions of the arterial tree (arrows), indicating extremely prolonged arterial transit time and not eligible for study enrollment.

The ASL perfusion imaging was constructed by the following images: one image taken shortly after the inflowing arterial spins were inverted (i.e. label image), another taken without inverting the arterial spins (i.e. control image) and the third one was the reference image used to quantify the CBF. Subtracting the label image from the control image produced an ASL perfusion-weighted image, which was then converted to a quantitative image that reflected CBF. For each subject, the CBF map was calculated using a scanner console with FuncTool 3DASL (DiscoveryMR450, GE Healthcare, Milwaukee, WI) within 1 minute. The signal is reported as mL/100 g/minute. The ASL-derived perfusion maps for each participant were carefully checked by an experienced neuroradiologist (C.C. Lui) to ensure the imaging quality was in accordance with the recommendation of the consensus^30^. These parameters included the scanner related artifacts, motion artifact or whether there was insufficient labeling due to prolonged arterial transit time. The images that were not qualified were excluded from the study and the represented images are shown in Fig. 1.

#### ECD-SPECT images

All subjects were injected intravenously with a single bolus dose of 110 MBq (30 mCi) 99mTc-ECD. Brain SPECT/CT (Symbia T; Siemens, Erlangen, Germany) images were obtained 30 minutes later. The SPECT/CT scanner was equipped with low-energy, high-resolution collimators and a dual-slice spiral CT. The acquisition parameters for SPECT were a 128 × 128 matrix with 60 frames (40 s/frame), and the scan parameters for CT were 130 kV, 17 mA, 5-mm slices, and image reconstruction with a medium-smooth kernel. The SPECT images were attenuation-corrected based on the CT images and scatter-corrected with the Flash 3D algorithm (ordered subsets expectation and 3D maximization with resolution correction) with eight subsets and eight iterations.

### Study scheme

Neuroimaging comparisons were processed using Spatial Parametric Mapping Version 8 software (The Wellcome Department of Imaging Neuroscience, London, UK) and customized MATLAB scripts (The Mathworks Inc., Natick, MA) with study-specific templates. In order to detect AD-related atrophy and hypoperfusion patterns, voxel-wise statistics were performed by comparing differences in 3DT1, ASL-MRI, and SPECT images between the patients and controls. The results were thresholded at p < 0.005, with false discovery rate correction for multiple comparisons and a cluster threshold of 200 voxels. Detailed pre-processing steps for each image type are listed below.

#### Structural image pre-processing

The 3DT1 images were processed using voxel-based morphometry version 8^31^. All 3DT1-weighted images were spatially normalized into the standardized Montreal Neurological Institute (MNI) space using a 12-parameter affine transformation and non-linear normalization, followed by re-slicing onto a voxel size of 1 × 1 × 1 mm to minimize partial volume effects. The images were segmented into GM and white matter (WM) compartments with intensity inhomogeneity correction, and modulated with Jacobian determinants to compensate for volume changes in non-linear spatial normalization. The segmented images were then smoothed with a 8 × 8 × 8-mm full-width at half-maximum isotropic Gaussian kernel. The normalized and smoothed GM probability maps were entered into a two-sample t test with age and years of education as covariates to detect changes in GM volume in the patients with AD.

#### ASL-MRI image pre-processing

Because of low resolution of the CBF images, we corrected the partial volume effect to improve the accuracy of CBF quantification. High-resolution MR image was segmented into GM, WM and cerebrospinal fluid (CSF) probability maps. GM and WM probability maps were then smoothed with a 1.88 × 1.88 × 4 mm^3^ kernel to mimic the PVE of the ASL images. The smoothed GM probability maps were then subsampled to the spatial resolution of the CBF images and thresholded at >0.3. Intensities of CBF images were corrected according to the following equation: I_corrected_ = I_uncorrected_/(P_GM_ + 0.4 * P_WM_)^32^, where the 0.4 factor is the perfusion ratio between WM and GM, and P_GM_ and P_WM_ are the probabilities of GM and WM, respectively. The PVE-corrected CBF images were spatially normalized to MNI space, re-sliced onto a voxel size of 1 × 1 × 1 mm^3^ and smoothed using a Gaussian filter with full-width at half-maximum of 8 mm. To eliminate outliers in the perfusion image confounded by large blood vessels or image processing computations, the threshold was set at a low value threshold of zero and a high-value threshold of 2 standard deviations above the mean perfusion for each subject^33^. The relative CBF (rCBF) maps were calculated by dividing each image by its whole brain CBF, and the rCBF in each regions of interest was used for statistical comparisons. The rationale not to use the absolute CBF for regional comparisons was based on the reports by Aslan *et al*. and Chen *et al*.^34,35^ that the use of rCBF may greatly reduce the variations of inter-subject physiologic noise. A two-sample t test with age and years of education as covariates was used to detect regional rCBF changes in the patients with AD.

#### ECD-SPECT image pre-processing

SPECT perfusion maps were corrected for PVE as described in previous study^36^. Briefly, high-resolution MR image was segmented into GM and WM probability maps and coregistered to the corresponding SPECT images and re-sliced to the same voxel size. These re-sliced GM and WM images were then convoluted with a 7 mm FWHM isotropic Gaussian kernel. WM-SPECT image was obtained from the multiplication of the WM maps by the mean SPECT count for areas more than 95% in WM concentration. A GM-SPECT image was obtained by subtracting the WM-SPECT image from the SPECT image. Finally, the GM-SPECT image was divided by the GM-MR image, yielding a PVE-corrected SPECT image. The PVE-corrected SPECT images were spatially normalized to the MNI template, re-sliced onto a voxel size of 1 × 1 × 1 mm^3^ and smoothed using a Gaussian filter with full-width at half-maximum of 8 mm. For count normalization, we used the cerebellum as the reference^37^. SPECT PI maps were obtained by dividing the image by the mean uptake in the cerebellum (counts/pixel). A two-sample t test with age and years of education as covariates was used to detect regional PI changes in the patients with AD.

#### Regions of interest analysis

To validate the results by voxel-based analysis, several regions of interest highly characterized in the patients with AD were defined in accordance with the automated anatomic labeling template^38^ and arterial transit time based flow territories developed by Mutsaerts *et al*.^39^. They included the frontal (but excluding the primary motor cortex), lateral temporal, MTC, parietal, occipital, middle frontal cortex, angular, PCC, precuneus, precentral motor cortex and cerebral cortex in distal territory of posterior cerebral artery (PCA). The MTC referred to the hippocampus, para-hippocampus and amygdala^38^. The lateral temporal region of interest referred to the superior, middle and inferior temporal areas, and the superior and middle temporal pole areas.

### Statistical analysis

All data were expressed as mean ± standard deviation. We used the Student’s t test for continuous variables and the chi-square test for categorical variables for comparisons between the patients and controls. In order to understand the effect of MTC atrophy on remote hypoperfusion, a linear regression model with adjustments for possible confounding covariates was used to evaluate the relationship between MTC volume and regional rCBF. Furthermore, we evaluated the relationships between regional PI and their corresponding GM volume by simple Pearson’s correlation, followed by multivariate linear regression analysis to test the independent effect of PI. Because MTC atrophy is a structural hallmark in AD and strongly associated with disease severity, we use MTC volume as one of the confounders in the multivariate linear regression analysis. The rCBF and PI images were entered into the linear regression model separately, which was adjusted for possible covariates, to evaluate associations with CASI total, short term memory, and language composite subdomain scores. Regression results were statistically thresholded at p < 0.05, with false discovery rate correction for multiple comparisons and a cluster threshold of 200 voxels. Pearson correlation analysis was used to test the relationships among cerebrovascular risk biomarkers and regional rCBF or PI, followed by multivariate linear regression analysis to test the independent associations. All statistical analyses were conducted using the Statistical Package for Social Sciences software package (SPSS version 22 for Windows®, SPSS Inc., Chicago, IL). Statistical significance was set at p < 0.01.

### Ethics approval and consent to participate

This study was conducted in accordance with the Declaration of Helsinki and was approved by the Institutional Review Board of Chang Gung Memorial Hospital (IRB 103-7745A3 and IRB 102-1298A3). The study participants and their caregivers agreed on the participation of the study with written informed consent.

### Availability of data and material

The datasets generated during and/or analyzed during the current study are not publicly available due to the intelligence rights owned by the hospital and the authors but are available from the corresponding author on reasonable request.

## Results

### Characteristics of the subjects

Table 1 shows the data of the patients and controls. The patients had a higher prevalence of APOE4, fewer years of education, lower scores in the cognitive tests, and lower regional and global CBF values. Patients with AD showed significant lower absolute values of CBF in all predefined regions.

**Table 1:** Clinical data between Alzheimer’s disease (AD) patients and age-matched controls.

	AD (n = 50)	Control (n = 30)
Age (years)	73.32(8.40)	71.03(8.05)
Education (years)	5.3(4.51)*	8.5(5.22)
Gender (female, %)	33(66%)	18(60%)
Diabetes Mellitus, cases (%)	9(18%)	6 (20%)
Hypertension, cases (%)	29(58%)	17(56.7%)
APOE4 carrier, cases (%)	27(54%)*	6(20%)
High sensitivity C- reactive protein (mg/L)	2.94(3.53)	1.72(1.61)
Creatinine (mg%)	0.90(0.26)	0.91(0.25)
Total cholesterol (mg/dl)	195(37.8)	180(29.0)
Triglyceride (mg/dl)	116(59.4)	109(47.2)
High-density lipoprotein (mg/dl)	57.3(13.3)	55.7(16.7)
Low-density lipoprotein (mg/dl)	112(30.2)	104(29.3)
Mini-Mental State Examination	16.78(5.13)*	27.07(1.91)
CASI total scores	56.46(17.61)*	85.81(6.00)
Mental manipulation	4.4(3.16)*	8.4(1.59)
Attention	5.8(1.41)*	6.9(0.92)
Orientation	10.6(4.56)*	17.1(2.26)
Long term memory	7.4(2.75)*	9.6(0.81)
Short term memory	3.0(2.08)*	8.9(1.51)
Abstract thinking	6.8(2.40)*	9.1(1.63)
Drawing	6.3(3.42)*	9.3(1.06)
Language Composite scores	11.5(4.73)*	16.5(2.43)
Cerebral blood flow(ml/100 g/min)		
Global	40.4(9.76)*	47.1(7.13)
Frontal cortex	44.6(10.4)*	50.6(7.01)
Lateral temporal regions of interest	42.5(7.54)*	49.0(6.97)
Medial temporal cortex	40.0(9.23)*	43.8(5.29)
Occipital cortex	34.0(10.1)*	43.2(9.23)
Parietal cortex	37.9(10.7)*	46.2(8.61)

Data are presented as mean (standard deviation) or the number (frequency, %); *p < 0.01.

Abbreviations: CASI, Cognitive Abilities Screening Instrument; APOE4, Apolipoprotein E4.

Medial temporal cortex = hippocampus, para-hippocampus and amygdala.

Lateral temporal regions of interest = superior, middle and inferior temporal areas, and the superior and middle temporal pole areas.

### Comparisons of two perfusion imaging modalities in patients and controls

**Figure 2:**
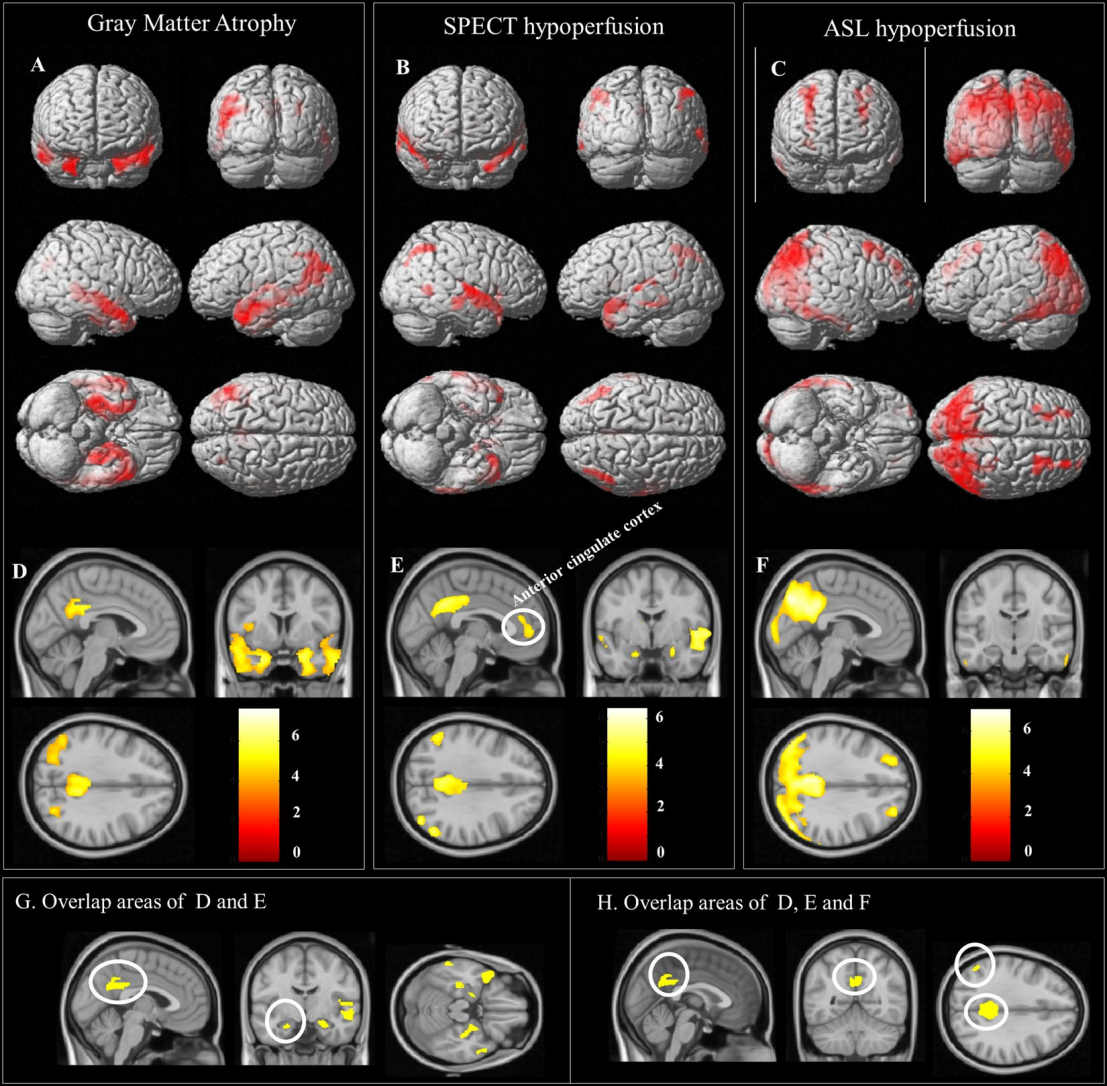
Voxel-wise statistical analysis between patients with Alzheimer’s disease (AD) and controls. Areas of (**A**,**D**) atrophy; (**B**,**E**) decreased perfusion index (PI); and (**C**,**F**) decreased relative cerebral blood flow (rCBF) in the patients. Figure **A**–**C**: rendered onto 3-dimensional brain images with color intensity representing the depth from the brain surface. Figure **D**–**F** are representative slices with a color bar indicating the t-value scale. The overlapping areas between lower PI and atrophy in AD are shown in Fig. 1G. The overlapping areas between lower PI, rCBF and atrophy in AD are shown in Figure H. All images were statistically thresholded at p < 0.05, and a false discovery rate correction for multiple comparison, cluster > 200. SPECT = single-photon emission computed tomography; ASL = arterial spin labeling; PCC = posterior cingulate cortex.

The results of voxel-wise statistical analysis between the patients and controls are shown in Fig. 2. Regions with decreased PI were found primarily in the angular gyrus, PCC, anterior cingulate gyrus, anterior and superior temporal lobe. Meanwhile, decreases of PI in the MTC were also found (Fig. 2B and E). For rCBF analysis (Fig. 2C and F), lower rCBF were more extended and found in the following areas (vessel territories)^39^: inferior temporal lobe (distal PCA territory); posterior parietal cortex and angular gyrus (middle cerebral artery [MCA]-PCA border zone areas); precuneus and posterior cingulate gyrus (MCA-PCA-anterior cerebral artery [ACA] border zone areas); and middle prefrontal cortex (ACA-MCA border zone areas). The coordinates of these regions are shown in Table 2.

**Table 2:** Summary of voxel-wise comparisons showing patients < controls.

Anatomic label	x, y, z (mm) coordinates			Z score	Voxels
Gray matter volume					
Right hippocampus	22	−33	−3	6.50	6966
Left hippocampus	−22	−18	−13	7.05	18241
Right middle temporal	51	−9	−18	7.06	4996
Right precuneus	6	−51	36	5.52	2195
Right middle occipital	28	−73	28	4.81	511
Relative cerebral blood flow (rCBF)					
Right angular	46	−69	42	7.02	11059
Right superior frontal	26	42	37	5.90	1939
Left superior frontal	−22	29	46	5.70	3246
Right middle frontal	27	14	43	5.53	4453
Left middle frontal	−34	13	57	5.41	800
Perfusion Index (PI)					
Left middle temporal pole	−30	13	−35	6.14	4248
Right superior temporal pole	52	5	−8	5.77	7584
Right caudate	13	22	8	4.84	1598
Left caudate	−11	7	11	6.10	2767
Left posterior cingulate	−4	−31	29	4.98	1224
Right angular	49	−61	51	4.95	1199
Left angular	−50	−61	48	4.45	526
Overlapping regions*					
Left posterior cingulum	−6	−43	24	6.09	3522
Left angular	−50	−65	41	4.71	1318
Right angular	42	−74	43	4.72	501

*Indicate overlapping areas in volume, rCBF and PI.

**Figure 3:**
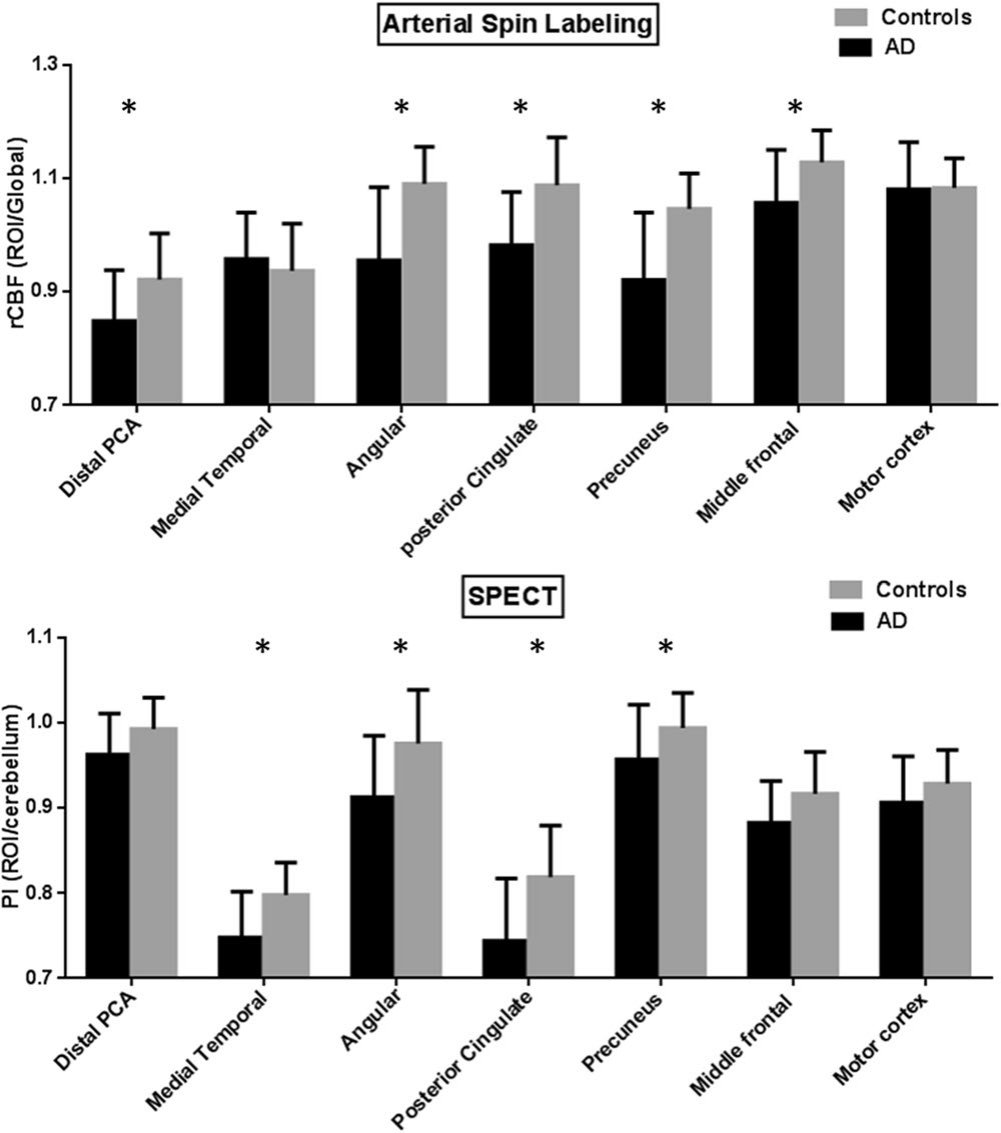
Comparisons of functional imaging parameters in the patients with Alzheimer’s disease (AD) and controls. (**A**) Relative cerebral blood flow (rCBF) by arterial spin labeling (ASL) and (**B**) perfusion index (PI) by SPECT from eight regions of interest. Error bars represent standard errors. PCA = posterior cerebral artery; SPECT = single-photon emission computed tomography. *p < 0.01.

The rCBF or PI signals from the pre-defined regions of interests were further extracted and compared between the patients and controls (Fig. 3). Combination with Figs 2 and 3, regions concurrently showing lower rCBF and PI in the patients were localized in the angular gyrus, PCC and precuneus gyrus, while the areas showing dissociations were seen in the superior temporal lobe and MTC (lower PI without lower rCBF) and distal PCA territory, inferior temporal lobe and middle frontal cortex (lower rCBF without lower PI). The perfusion in motor cortex did not show difference between AD and controls regardless the results of rCBF or PI.

### Comparisons of functional (perfusion) and structural images (volumes) in patients and controls

Regions of atrophy were primarily localized in the medial and lateral temporal structures (Fig. 2A), PCC and angular gyrus (Fig. 2D). The coordinates of these regions are shown in Table 2. The spatial similarities between lower PI and atrophy were higher than lower rCBF and atrophy. The overlapping areas of lower PI and atrophy are located in medial temporal lobe, PCC and angular gyrus which were shown in Fig. 2G, and the regions showing lower PI, lower rCBF and atrophy included the PCC and angular gyrus (Fig. 2H). Lower rCBF without atrophy was mainly located in inferior temporal lobe, part of parietal association cortex and middle frontal cortex. Atrophy without lower rCBF was mainly located in medial temporal lobe.

### The effect of MTC atrophy on remote rCBF

**Figure 4:**
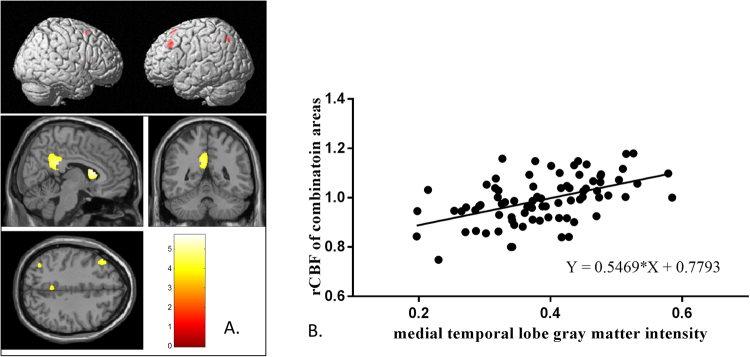
Relationships between relative cerebral blood flow **(**rCBF) and medial temporal volume. rCBF map showing positive correlation with medial temporal cortex (MTC) volume rendered onto 3-dimensional brain images, with color intensity representing the depth from the brain surface. Representative slices with a color bar representing the range of t values. Images were statistically thresholded at p < 0.05, and a false discovery rate correction for multiple comparison, cluster > 200. (**B**) Scatter plots showing linear correlation between the volume of MTC and rCBF of the combined signals from Fig. 2H.

The rCBF signals that corresponded to the MTC volumes included PCC, left angular gyrus, bilateral caudate and left middle frontal cortex (Fig. 4A). For regions that showed overlap between GM atrophy, lower PI and rCBF (Fig. 2H), the overall rCBF values were extracted and combined. The combined rCBF was significantly correlated with MTC volume (Fig. 4B, r^2^ = 0.243, p < 0.01).

### The relationship between regional perfusion and corresponding cortical volumes

The simple correlation showed regional PI were significant correlated with their corresponding cortical volumes (Table 3). After adjusted for age and MTC volumes, angular gyrus and precuneus volumes were still significant related to their own PI (for angular p = 0.008, for precuneus p = 0.006) (Table 3). The association in frontal lobe, occipital lobe, parietal lobe, middle frontal gyrus and PCC does not reach significance after adjusted for age and MTC volumes (p > 0.01).

**Table 3:** The relationship between regional PI and corresponding cortical volume.

	Simple correlation^+^	Adjusted correlation^#^
Frontal	0.456(0.000)*	0.126(0.077)
Lateral temporal	0.553(0.000)*	0.077(0.250)
Occipital	0.259(0.020)	0.055(0.464)
Parietal	0.375(0.001)*	0.161(0.061)
Middle frontal	0.421(0.000)*	0.108(0.144)
Angular	0.493(0.000)*	0.244(0.008)*
Posterior Cingulate Cortex	0.500(0.000)*	0.176(0.114)
Precuneus	0.414(0.000)*	0.244(0.006)*

^+^Simple correlation, the correlation between regional PI and corresponding cortical volume (ex. Frontal represented the association between frontal PI and frontal volume).

^#^Multivariate linear regression analysis, corresponding cortical volume were dependents and regional PI were independents and adjusted for age and medial temporal cortex volume.

Abbreviations: PI, perfusion index from SPECT images.

*p < 0.01; numbers indicate correlation coefficient (p-value).

### Clinical significance of the functional networks in ASL-MRI and ECD-SPECT

Correlation maps of rCBF or PI with CASI total and short-term memory subdomain CASI scores are shown in Supplementary Figures 2 and 3. The global CASI scores and short-term memory scores were positively correlated with PI in bilateral medial and lateral temporal lobes, PCC, precuneus, and angular gyrus. With regards to rCBF, significant regions were shown in the posterior parietal, inferior temporal, PCC, precuneus, and angular gyrus.

**Figure 5:**
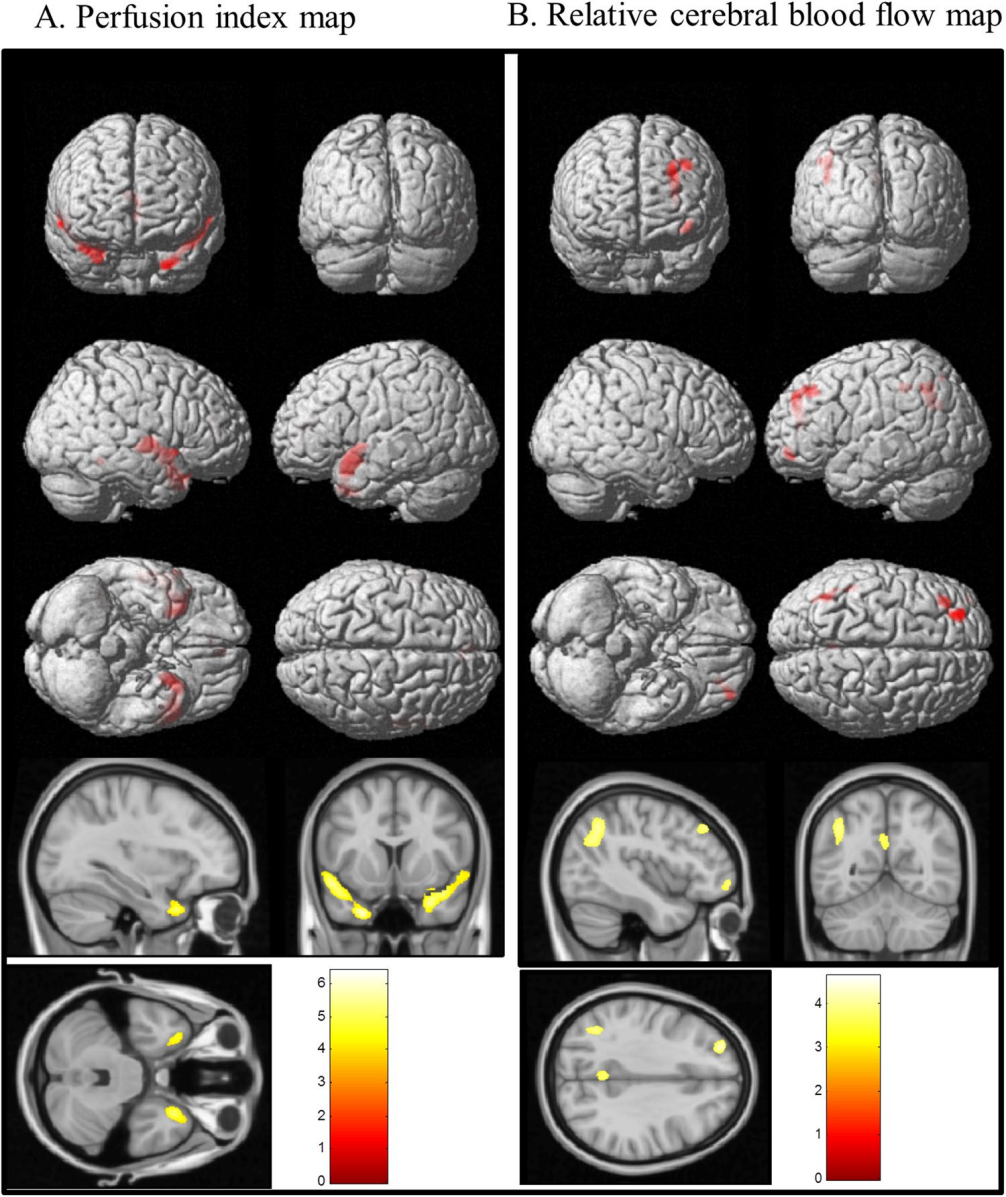
Correlation between composite language scores and (**A**) perfusion index map and (**B**) relative cerebral blood flow. The red color rendered onto the 3-dimensional brain images represents positive correlations with intensity as the depth from the brain surface. Representative slices with a color bar representing the range of t values are shown below. Images were statistically thresholded at p < 0.05, and a false discovery rate correction for multiple comparisons, cluster > 200.

Results of partial correlation between language composite score and PI (Fig. 5A) and rCBF (Fig. 5B) adjusted for age and education were lateralized to the left hemisphere. The language composite scores were correlated to PI in the anterior and superior temporal lobe (Fig. 5A) and the rCBF in the left angular gyrus, PCC and left-mid frontal gyrus (Fig. 5B).

### Cerebral vascular risk factors may affect regional rCBF and PI

Simple correlation analysis showed significant relationships between the level of HDL and rCBF in the PCC, even after adjusting for the severity of dementia and age (both p < 0.01) (Table 4). hsCRP was inversely correlated with frontal, lateral temporal, medial temporal and PCC PI (p < 0.01). After adjusting for age and CDR sum of box, the significant association remained in the lateral temporal and PCC regions (Table 4).

**Table 4:** Relationships between vascular risk factors and functional imaging parameters.

Regional of interests	HDL and rCBF		hsCRP and PI	
	Simple correlation	Adjusted correlation^#^	Simple correlation	Adjusted correlation^#^
Frontal	−0.03(0.817)	−0.25(0.313)	−0.34(0.002)*	−0.29(0.011)
Lateral temporal	−0.17(0.129)	−0.14(0.193)	−0.37(0.001)*	−0.29(0.006)*
Medial temporal	−0.08(0.481)	−0.54(0.625)	−0.30(0.007)*	−0.24(0.031)
Occipital	0.19(0.101)	0.16(0.122)	−0.15(0.199)	−0.08(0.470)
Parietal	0.10(0.389)	0.08(0.443)	−0.21(0.06)	−0.15(0.187)
Angular	0.03(0.777)	0.02(0.884)	−0.26(0.019)	−0.19(0.078)
Posterior Cingulate Cortex	0.30(0.007)*	0.28(0.006)*	−0.36(0.001)*	−0.28(0.007)*
Precuneus	0.15(0.187)	0.13(0.181)	−0.14(0.231)	−0.06(0.600)

Abbreviations: HDL, high-density lipoprotein; hsCRP, high sensitive C-reactive protein; rCBF, relative cerebral blood flow; PI, perfusion index.

^#^Adjusted for age and clinical dementia rating sum of box.

*p < 0.01; numbers indicate correlation coefficient (p-value).

## Discussion

### Major findings

Based on the multiparametric imaging modalities, this study tested the inter- relationships between perfusion status and GM atrophy. There were four major findings. First, both ASL-MRI and ECD-SPECT showed low perfusion in the angular gyrus and PCC. Regions showing dissociation in two perfusion modalities were maily localized in the lateral temporal, medial temporal and occipital. The dissociation patterns may reflect the differences of tracer properties in physiological dynamics. Second, we observed significant linear relationships between the MTC GM volume and rCBF of PCC and angular gyrus. With previous pathological^40–42^ and longitudinal studies^43^, we purposed possible mechanisms to support the link between MTC atrophy, watershed hypoperfusion and watershed atrophy. Third, regional cortical perfusion in the superior and anterior temporal lobe areas in PI image and left MCA watershed in rCBF image is associated with language scores. Fourth, our results suggest that an elevated level of hsCRP and a decreased level of HDL are serum risk biomarkers for cerebral hypoperfusion. Although both ASL-MRI and ECD-SPECT provide information on cerebral perfusion, the differences in regional perfusion patterns and vascular risk factors highlight the different mechanisms and clinical perspectives of these two types of perfusion scan.

### Dissociations in two perfusion modalities

Our study is the first to compare ECD-SPECT and ASL-MRI perfusion pattern in AD. We found dissociations in regions showing lower CBF (posterior parietal and inferior temporal lobe) and PI (medial and superior temporal lobe). The regions showing dissociations in two perfusion modalities may implicate differences in tracer uptake mechanisms.

Signals of ECD-SPECT often reflect the microvasculature status as the retain of Tc99m-ECD in the brain parenchyma are rapid and the clearance is slow^13^. Based on the tracer properties, the half-life of ECD in the brain parenchyma is long enough to allow the tracer to saturate within the microvasculature environment so the acquisition time can be at set 1/2 hour or later. In physiological conditions, regional cortical activation depends on the demand of glucose and the blood flow^13^. Therefore, lower PI in our AD patients may reflect decreases of metabolic demands due to GM atrophy and disturbance of the microvasculature circulation. The topographic relationships between MTC GM atrophy and local or distant region PI were observed in our study that may support the aforementioned mechanisms.

In contrast, ASL-based perfusion measurement involves a race between the decay of the spins and the delivery of labeled blood to the tissue^30^. Previous study^44^ showed that flow velocity related to macro-circulation and impairment of vascular autoregulation could influence CBF patterns. Mutsaerts *et al*.^39^ also suggested that CBF without vessel suppression reflected macro- and micro-vascular compartment. As the accentuation of signals in ASL-MRI provide additional information of the macro-circulation^45^, we purposed that areas with rCBF/PI dissociations (lower rCBF but without lower PI) were related to macro-circulation insufficiencies. The posterior parietal and inferior temporal lobe showing low rCBF were located in the distal PCA territory and middle frontal cortex was located in the MCA-ACA watersheds. These findings were consistent with our theory.

### Cerebral hypoperfusion related to global or distant region atrophy

Mismatch of cerebral hypoperfusion and GM atrophy in AD has been reported in studies using SPECT^43^, PET^46^ and perfusion MRI^47,48^. The most consistent finding across several ASL perfusion studies of AD is decreased CBF in PCC, precuneus and posterior temporoparietal cortex^9,49^. As the disease progress^43^, the hypoperfusion can extend to parieto-occipital cortex, inferior temporal^9^ and middle frontal cortex^35^. In contrast, medial temporal lobe atrophy is more an early imaging biomarker in AD and more profound than the anterior and superior temporal lobe^43^. The mismatch of topography in ASL image and atrophy were similarly observed before^47^. As the study population and enrollment criteria may not be the same across different studies, the small inconsistency may reflect differences of demographic data and disease severities. One ASL perfusion study^12^ suggested that the hypoperfusion pattern may be served as a diaschisis indicator of MTC damage. Another perfusion study^48^ suggested the phenomenon be related to functional disconnection. Current study directly reported significant correlations between MTC volume and rCBF at angular gyrus, PCC and precuneus so the structural-functional inter-relationships of the mismatches were validated. However, we speculate that these areas are located in the watersheds^50^, hence they are vulnerable to the flow insufficiency.

### Cerebral hypoperfusion related to vascular insuffiencies by disease pathology

By transcranial Doppler, smaller hippocampal volumes can lead to a decrease of CBF velocity^51^. As β-amyloid deposition in MTC areas may lead to volume atrophy and reduce the demand of CBF from PCA, PCA watershed areas are the most vulnerable regions if small vessel disease or impairment of autoregulation coexisted. Because ASL perfusion MRI was sensitive to the changes of macro-circulation, as disease progress the decrease in distal PCA blood flow would affect the rCBF to the posterior parietal and inferior temporal lobes.

Comparing the topographies of reduced PI and GM atrophy in our AD group, the spatial extent overlapped considerably in the posterior parietal association cortex, and PCC which is in agreement with previous reports^43,52^. An interesting finding was that MTC showed a small decrease in PI with or without PVE. Based on the physiological nature of SPECT, the PI results may have been confounded by volume atrophy and neuronal integrity^36^. However in one investigation, a mismatch of hypoperfusion and atrophy was found in very early stage AD^43^. Depending on the status of the compensatory mechanism, perfusion^47^ or metabolic rate^53^ in AD may initially increase and then subsequently decrease. Increased, nearly normal and reduced PI values have been reported in the MTC areas in patients with mild cognitive impairment and AD^47,54^. Although we enrolled patients with early-stage AD (CDR scores of 0.5 or 1), most of the patients may have initiated decompensatory processes in view of the decreased PI.

### Abnormal perfusion related to local atrophy

In normal health brain, regional perfusion is associated with the activity of neurons. Activated neurons have increased glucose consumption, but have only a limited ability to store glucose^55^. Therefore, increased cerebral blood flow is needed to deliver the glucose required for increased metabolic needs so CBF is coupled to neuronal activity^55^. However, in pathologic brain, the causal effect between hypoperfusion and brain atrophy were more complicated. A few studies focus on the relation between global CBF and total brain volume. One ASL-MRI study suggests adverse effects of reduced cerebral perfusion on brain structure in older adults with cardiovascular disease^56^. Another ASL-MRI study^57^ concluded that lower CBF reflects disease burden of both neurodegeneration and small vessel disease in AD. In the longitudinal study^42^, one study suggested brain atrophy causes CBF to decrease over time, and only in persons aged >65 years of age with the impairment of autoregulatory, hypoperfusion may have the adverse effect on brain volume. In the current study, we delineated the relationships between perfusion in angular gyrus and precuneus and their corresponding cortical volume which is independent to MTC volume and age.

### Confounding factors for watersheds perfusion changes

Although the cross-sectional design of the current study precludes interpretation of directionality, such findings raise the possibility that cerebral hypoperfusion is a significant contributing factor to adverse brain changes. In patients with^40^ and without AD^41^, pathological studies have provided evidence linking cerebral hypoperfusion and cortical watershed microinfarcts. Neurovascular units include perivascular neurons, astrocytes and vascular endothelial cells that control cerebral autoregulation to ensure sufficient CBF for daily metabolic needs^58^. Individuals with cerebral vascular risk factors often have dysregulated autoregulation and their neurons may be vulnerable even in physiological conditions such as changing posture^59^. Cerebral hypoperfusion in AD can be complex, and factors including aging, cerebral vascular risk factors^3^, β-amyloid deposition, endothelial dysfunction and autoregulation deficits^60^ may interact with each other to differing degrees to create a critically attained threshold that triggers microcirculatory disturbances, neuronal damage and atrophy^4^. As the angular gyrus and precuneus are located in the medial or lateral part of the major vessels watersheds^50^, their neurons may be vulnerable to slight macro-circulation change with impairment of autoregulation.

### Effects of cholinesterase inhibitors on perfusion patterns

In our AD patients, they were treated with cholinesterase inhibitors. Claassen *et al*.^61^ proposed Cholinergic–Vascular Hypothesis from experiments, preclinical and clinical evidence. They suggested the effect of cholinesterase inhibitors on CBF is not only from increased metabolism but also direct vasodilatation. In clinical practice, this group of medication also showed the benefit to patients with vascular dementia^62^. In current study, since cholinesterase inhibitors would diminish the significance of hypoperfusion in AD, we suggest more severe hypoperfusion would be found in AD without treatment. Besides, ameliorate circulation through cholinesterase inhibitors and then improve the clinical symptoms seems to imply the importance of cerebral perfusion in AD. However, with the disease progression, β amyloid resulted in total brain toxicity and finally can’t compensate from increase blood flow.

### ASL-MRI as a putative neuronal injury biomarker

We performed correlation analysis between different profiles of cognitive scores and PI or rCBF. Of note, the rCBF signals in the angular gyrus, PCC and middle frontal gyrus reflected predominantly left-hemisphere language composite scores, and these regions are localized in the left MCA watershed territory^50^. The topography correlated with cognitive scores in ASL-MRI may reflect the putative role of ASL-MRI as a neuronal injury biomarker in patients with AD without major vascular stenosis. The correlation pattern was different from the ECD-SPECT analysis in anterior and superior temporal lobes. The relationships between language networks may be inconsistent due to the different methodologies, however the networks emphasize the importance of the temporal, inferior parietal and posterior frontal lobes.

### Low HDL and elevated hsCRP may predict intracerebral hypoperfusion

HDL and hsCRP were both important biomarkers for predicting cerebral perfusion status in this study. The link between cholesterol and atherosclerosis has been well established from epidemiologic and statin therapy studies, while a reduced HDL level is a known risk biomarker for cardiovascular disease^63^, and it has also been correlated with cognitive function^64^ and GM volume^65^. In our ASL-MRI study, the modulation of HDL and cerebral perfusion were identified in the PCC area.

hsCRP is an important biomarker for vascular insufficiency^66^ that reflects non-specific inflammatory processes. The Bogalusa Heart Study^67^ reported that hsCRP is an independent predictor for carotid artery intima-media thickness, and the Rotterdam Study^68^ suggested that hsCRP is a mediator of cerebral small-vessel disease. However, our study is the first to identify an association between hsCRP level and reduced cerebral perfusion. Although indirect, amyloid toxicity may reflect a chronic low-grade inflammatory state that is related to the elevated hsCRP and lower PI shown in ECD-SPECT. It is worth noting that the significance of the correlation between hsCRP and PI decreased after adjusting for the severity of dementia and age. As both factors reflect general measurements of GM atrophy, these findings again reflect the confounding effect of brain volume in ECD-SPECT signals.

### Hypoperfusion-Atrophy hypothesis in AD

**Figure 6:**
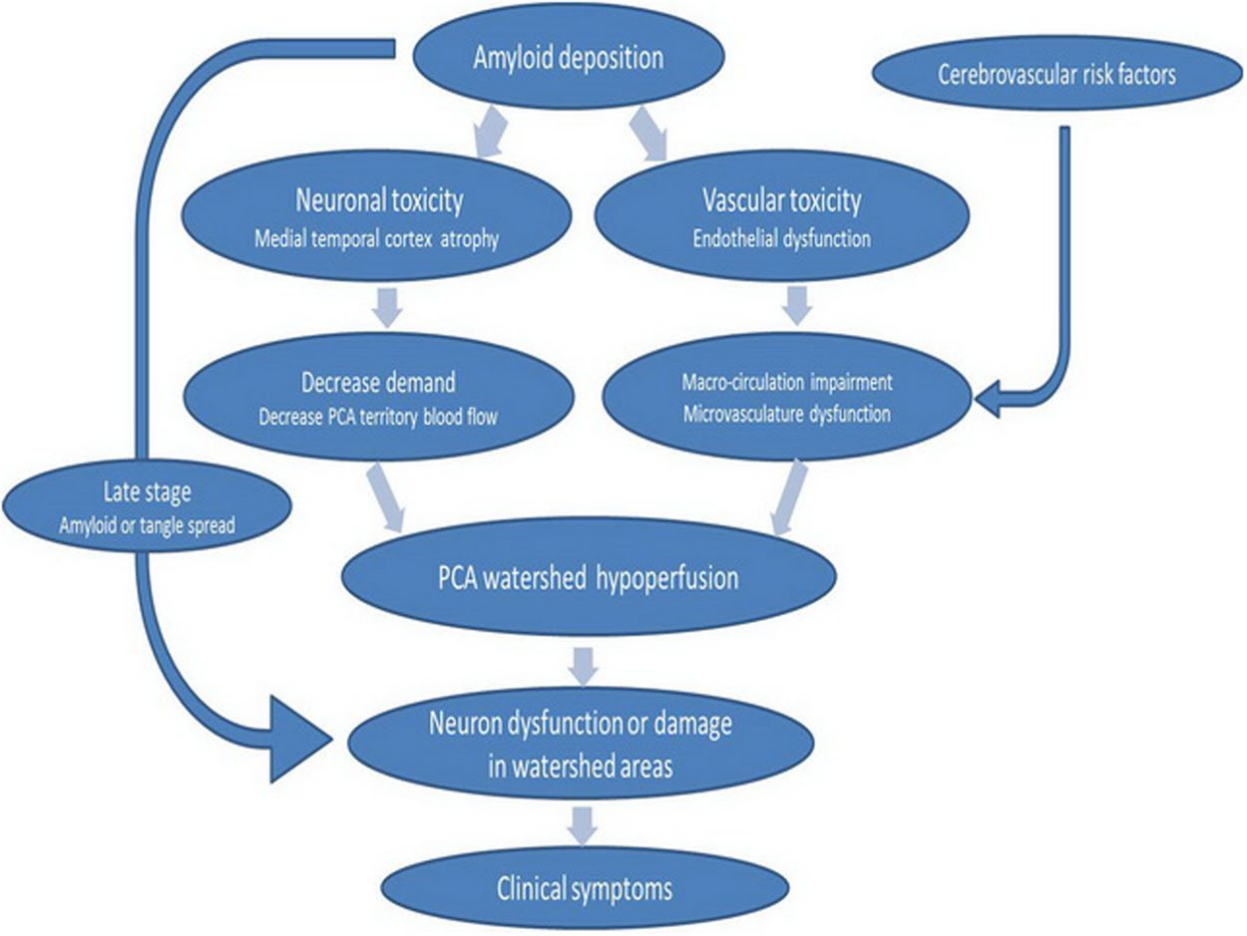
Hypoperfusion-Atrophy hypothesis in AD. The diagram shown our hypothesis about remote effect of medial temporal cortex atrophy on watershed perfusion and watershed hypoperfusion resulted in neuronal dysfunction and finally lead to clinical symptoms. Macro-circulation referred to global or territory blood flow. Microvasculature integrality included neuron, small vessels and autoregulation.

Figure 6 concluded our hypothesis about the relationship between hypoperfusion and atrophy in AD. In early stage of AD, amyloid deposited in MTC and vessels resulted in neuronal and vascular toxicity. Decreased demand due to atrophy and endothelial dysfunction lead to watershed areas hypoperfusion. Watershed hypoperfusion might influence neuronal function thus causing clinical symptoms. In more aggressive stage of AD, amyloid and tau spread extent which exacerbates the neuronal damage and makes clinical symptoms rapid deterioration. In addition to amyloid related vascular toxicity, other cerebrovascular risk factors including higher hsCRP and lower HDL also caused disturbances of macro- or microvasculature circulation.

### Limitations

There are several limitations to this study. First, the sample size in our study was small so our results may not represent all AD patients. Nonetheless, the pattern of cerebral hypoperfuson in this study is similar to previous reports in both SPECT and ASL-MRI studies. Second, the mechanisms between CBF and cortical atrophy are complex and the cross-sectional study design was not able to evaluate the top-down relationships. Further longitudinal follow-ups and experiment studies are needed to validate our observations. Third, the discussion of β-amyloid-related endothelial injury was based on the amyloid toxicity theory. Further analysis incorporating amyloid images may help to delineate the threshold levels as confounded by amyloid load. Lastly, although ASL images were carefully checked and we already excluded those with moderate to severe artery stenosis, mild degrees of labeling insufficiency could still happen. CBF without crushing could retain the signal-to-noise ratio^30^ and provide more information about macro-vascular circulation^39^. However, to understand the patterns in patients with vessel stenosis or advanced staging, the consideration of performing multiple post-label delay time and vascular crushing gradients will be necessary to reduce labeling insufficiency and remove the noise in large artery for a more accurate microvasculature CBF value.

## Conclusion

In conclusion, this multi-parametric neuroimaging analysis of AD explored the hypoperfusion patterns and delineated the importance of watershed territories. Features of ASL-MRI and ECD-SPECT provided complementary information to structural images. The ASL-MRI patterns reflected macro- and microvasculature circulatory and the ECD-SPECT patterns reflected microvasculature perfusion and tissue compartment. We hypothesize the effect of MTC atrophy on remote hypoperfusion in PCA watershed is through the decrease of PCA flow and impairment of cerebral autoregulation. And then watersheds hypoperfusion may be associated with regional atrophy. However, this hypothesis need further longitudinal and experiment study to prove.

## Electronic supplementary material

Supplementary information 
