# Supplementary material for: Cerebral Perfusion Insufficiency and Relationships with Cognitive Deficits in Alzheimer’s Disease: A Multiparametric Neuroimaging Study
Source: Sci Rep. 2018 Jan 24;8:1541. doi: 10.1038/s41598-018-19387-x (PMC5784155; doi:10.1038/s41598-018-19387-x)
Supplement: Supplementary file 1 — Supplementary information [file 41598_2018_19387_MOESM1_ESM.pdf]

Cerebral Perfusion Insufficiency and Relationships with Cognitive Deficits in Alzheimer's Disease: A Multiparametric Neuroimaging Study

Chi-Wei Huang[1], Shih-Wei Hsu[2], Ya-Ting Chang[1], Shu-Hua Huang[3], Yung-Cheng Huang[3], Chen-Chang Lee[2], Wen-Neng Chang[1], Chun-Chung Lui[4], Na-Ching Chen[1], Chiung-Chih Chang[1]

[1]Department of Neurology, Cognition and Aging Center, Kaohsiung Chang Gung Memorial Hospital, Chang Gung University College of Medicine, Kaohsiung, Taiwan
[2]Department of Radiology, Kaohsiung Chang Gung Memorial Hospital, Chang Gung University College of Medicine, Kaohsiung, Taiwan
[3]Department of Nuclear Medicine, Kaohsiung Chang Gung Memorial Hospital, Chang Gung University College of Medicine, Kaohsiung, Taiwan
[4]Department of Radiology, Division of medical imaging, E-Da Cancer Hospital and I-Shou University, Taiwan

Correspondence to: Chiung-Chih Chang, M.D., Ph.D.
Department of Neurology, Kaohsiung Chang Gung Memorial Hospital and Chang Gung University College of Medicine, #123, Ta-Pei Road, Niaosung, Kaohsiung County 833, Taiwan
Telephone: +886-7-7317123-3389
E-mail: neur099@adm.cgmh.org.tw

Supplementary

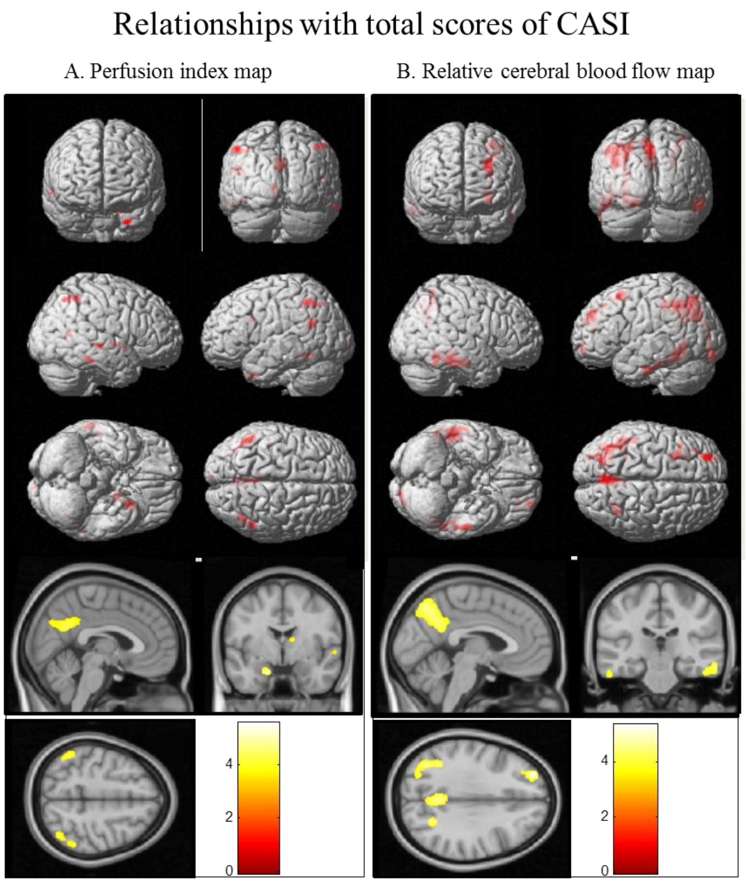

Figure 1. Correlation map between total scores of the Cognitive Ability Screening Instrument (CASI) and (A) perfusion index and (B) relative cerebral blood flow. The red color rendered onto the 3-dimensional brain images represents positive correlations and the intensity represents the depth from the brain surface. Representative slices with a color bar representing the range of t values are shown below. Images were statistically thresholded at $p<0.05$, and a false discovery rate correction for multiple comparisons, cluster>200.

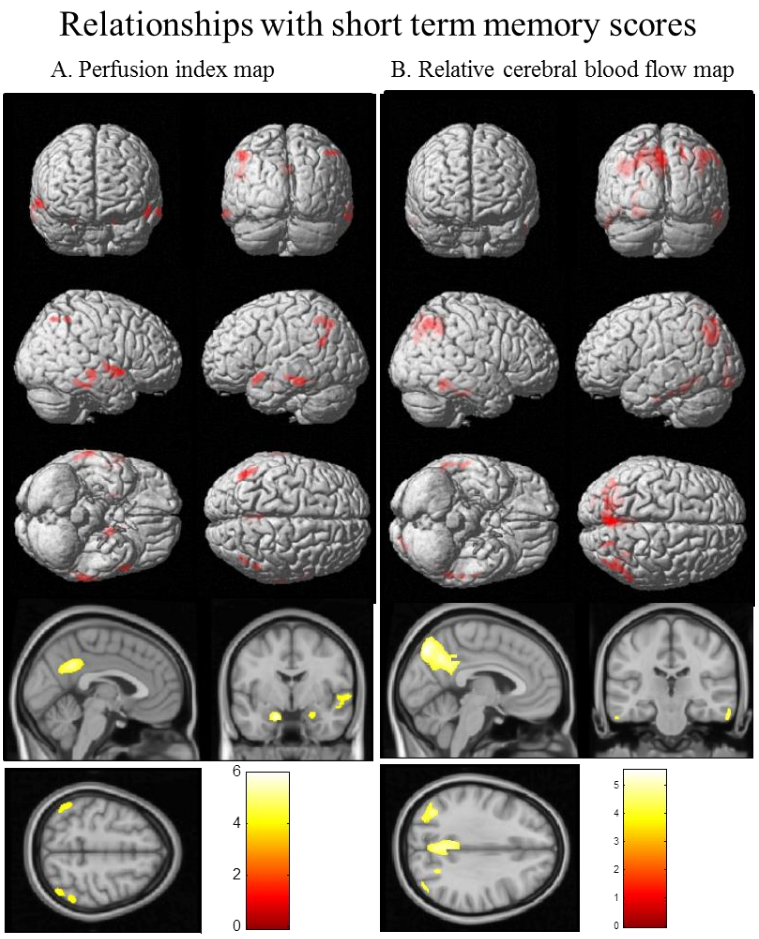

Figure 2. Correlation map between short-term memory scores of the Cognitive Ability Screening Instrument and (A) perfusion index and (B) relative cerebral blood flow. The red color rendered onto the 3-dimensional brain images represents positive correlations and the intensity represents the depth from the brain surface. Representative slices with a color bar representing the range of t values are shown below. Images were statistically thresholded at $p<0.05$, and a false discovery rate correction for multiple comparisons, cluster>200.

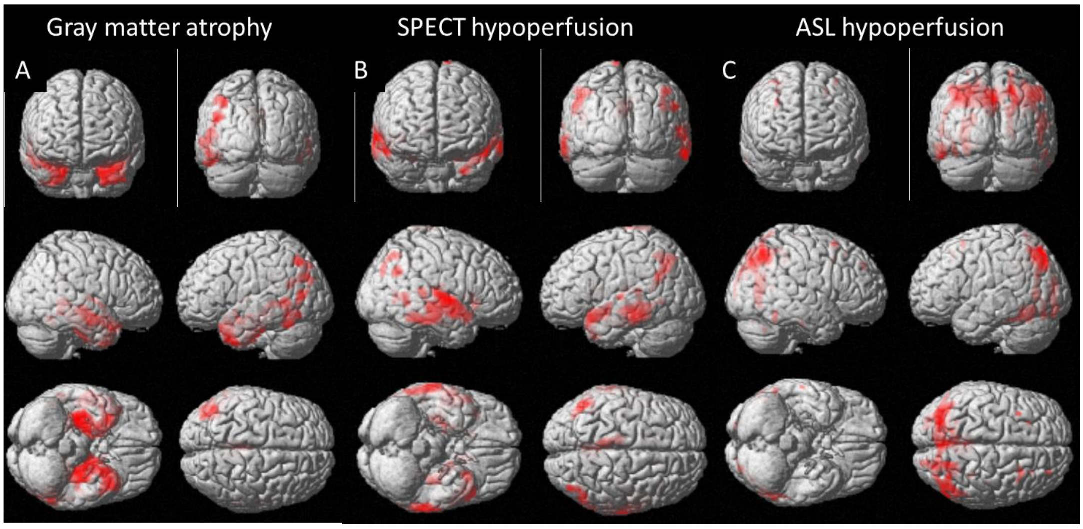

Figure 3. Voxel-wise statistical analysis using statistical nonparametric mapping (SnPM) between patients with Alzheimer's disease and controls.

Areas of (A) atrophy; (B) decreased perfusion index; and (C) decreased relative cerebral blood flow in the patients. The results are similar to statistic parametric mapping (SPM) analysis. All images were statistically thresholded at p <0.05, and a family- wise error rate correction for multiple comparison, cluster>200. ASL=arterial spin labeling; SPECT=single-photon emission computed tomography;
